# Educational and Behavioral Models for Promoting Health in Saudi Arabia: A Theoretical Overview

**DOI:** 10.7759/cureus.9471

**Published:** 2020-07-30

**Authors:** Abdulaziz M Alsufyani, Khaled E Almalki, Abeer Almutairi, Sayer M Aljuaid, Bandar O Alsufyani

**Affiliations:** 1 Comprehensive Rehabilitation Center, Ministry of Human Resources and Social Development, Taif, SAU; 2 Primary Health Care Center, Ministry of Health Holdings, Riyadh, SAU; 3 Ministry of Health, King Fahad Specialist Hospital, Buridah, SAU; 4 Primary Health Care Center, Ministry of Health Holdings, Taif, SAU

**Keywords:** behavioral models, ecological theory, educational models, health education, health promotion, saudi arabia

## Abstract

In recent years, the field of environmental health promotion gained new prominence as awareness of physical environmental stressors and exposures escalated in Saudi Arabia. Although, several theories and conceptual models are routinely used for guiding health education and promotion interventions, they are rarely applied to environmental health issues in Saudi Arabia. This study theoretically reviews the educational and behavioral models for promoting health in Saudi Arabia. This study examines how education and behavioral models can reduce exposure to environmental health risks. In particular, this article describes the application of ecological theory with regard to its theoretical, analytical, and methodological challenges to future research in educational and behavioral studies. The implications of ecological theory are discussed for environmental health promotion. Ecological theory can further demonstrate the dimensions of health behaviors such as procedures for changing them. Health behaviors are also affected by many forces such as physical and social environments. This theory can assist planners in identifying the most appropriate target audiences, methods to improve change, and consequences for assessment. This theory is also important in including social, cultural, and physical factors that affect health consequences, which include factors such as social cohesion, behavioral patterns, emotional wellbeing, genetic heritage, and developmental maturation.

## Introduction and background

Health promotion offers opportunities for both individuals and communities to obtain essential information skills needed to change health behaviors and make healthy decisions [1]. In Saudi Arabia, the field of environmental health promotion has gained new prominence due to the escalation in awareness of physical environmental stressors and exposures. The government of Saudi Arabia attempts to enhance healthcare using health education and promotion, and this remains an emphasis on healthcare policies [2]. Therefore, in this theoretical paper, an overview of healthcare systems and concept of health promotion will be discussed in the context of Saudi Arabia.

## Review

Overview of healthcare systems and health promotion

An important role has been played by the government of Saudi Arabia to manage and develop the healthcare sector. During a five-year development program, in 1970, the government implied strategies for quickly expanding and improving its healthcare system [3]. For example, the number of hospitals increased from 74 to 350 over a 35-year span. The healthcare services are funded by the government, and the Ministry of Health (MOH) provides healthcare and organizes its different services along with several semi-publicly funded organizations. This framework provides healthcare services to all citizens with the combined use of public and private hospitals. The government has created a two-tier health service phase for implementing this system. A network of primary healthcare centers and clinics within the country is the first-tier [4].

In Saudi Arabia, healthcare services have been highly prioritized by the government. Recently, health services have enhanced majorly with respect to quality and quantity. Saudi Arabia has a large geographic expanse and population and thus made accessible high-level of care virtually to all segments of the population as compared to other nations of the world [5]. The first public health department, based on a royal decree from King Abdul-Aziz, was established in Mecca 1925. The responsibility of this department was to sponsor and monitor free healthcare for the pilgrims and population by establishing a number of dispensaries and hospitals [6]. The development of the MOH in 1950 was another important step under another royal decree. The government has introduced five-year development plans, 20 years later, to enhance all sectors of the Kingdom, which include the most dominant Saudi healthcare system [7].

The progression in healthcare services has contributed to the significant enhancements in health determinants along with other factors, which include better life conditions, more accessible public education, and improved health awareness. However, there is no clear communication or coordination platform among them, which results in a waste of effort and resources despite the diversity of health service providers [8]. For instance, there are significant opportunities for taking advantage of laboratories, training programs, well-trained individuals, and equipment from different nations. On the contrary, the benefit of these opportunities is restricted throughout each sector due to poor coordination. A royal decree established the Council of Health Services to overcome this situation and to offer the population with affordable, equitable, updated, organized, and comprehensive healthcare [9]. Significant progress has yet to be achieved in this health sector, even though the objective of the Council was to establish a policy for integration and coordination among all healthcare service authorities [10].

Transition to primary healthcare center (PHC) services

Health services in Saudi Arabia were majorly curative and emphasize the provision of treatment for current health problems in line with the anticipations of the population [11,12]. However, the curative care model can be expensive to health providers, when the prevention or minimization of a number of diseases is in-line using a preventing strategy. The MOH executed a variety of preventive measures using former health offices, child health care, and maternal centers. Likewise, vertical programs such as tuberculosis, leishmaniasis control, and malaria were performed for controlling the activities of a disease [12].

The establishment of appropriate premises was the first step within the country. Current facilities situated in adjacent areas were combined into single units. These units include maternal and child health centers, former health offices, and dispensaries. In small and rural districts, the health posts were upgraded to PHC centers. The focus of the health centers is on the eight major aspects of the PHC approach, which include the methods of preventing and controlling them, educating the population concerning prevailing health problems, promotion of food supply and adequate nutrition, prevention and control of locally endemic diseases, immunization of children against major communicable diseases, provision of appropriate supply of safe water and basic sanitation, provision of essential drugs, provision of comprehensive maternal and child healthcare, and adequate treatment of common injuries and diseases [11].

The number of visits to outpatient clinics has been reduced by emphasizing on a PHC strategy and implementing a logical referral system [12]. During 2009, approximately 82% of client visits to MOH facilities were to PHC centers, which comprise more than 54 million PHC clients [9]. Inside each PHC center, the creation of individual and family health records has lowered the duplication of physicians. The costs of medications have not reduced by the use of essential drugs list and documentation of prescriptions inpatient health files.

Appropriateness of ecological theory in answering the problem

Ecological theory allows planners’ techniques to move beyond intuition to design and assess health promotion and health behavior interventions on the basis of behavioral understanding. It assists in seeking to step back and considering the larger picture. For instance, a program planner, using ecological theory, plans health interventions by creating innovative approaches to address particular conditions.

Ecological theory can be considered as a foundation for the planning and development of a program with the current focus on using evidence-based interventions in behavioral medicine, medicine, and public health. A road map is provided by the theory to study issues, creating adequate interventions, and assessing their gains [13]. It can inform the intuitions of a planner during all of these phases, providing insights that interpret into stronger programs. Ecological theory can further demonstrate the dimensions of health behaviors such as procedures for changing them. Health behaviors are also affected by many forces such as physical and social environments. This theory can assist planners in identifying the most appropriate target audiences, methods to improve change, and consequences for assessment.

Ecological theory is essential in public health education because of its social context in which behavior occurrence is always evolving. New social science research enables ecologists to refine and implement current theories at the same time. In particular, some constructs are important to multiple theories such as self-efficacy. Usually, a change in behavior is required by reduction or mitigation of exposure of an individual exposed by a policy maker who can ratify laws for reducing the exposure. In general, health promotion and health education theories and conceptual models are extremely important to guide interventions that require behavioral changes for reducing exposure to environmental hazards [13]. On the contrary, ecological theory is relatively used for guiding health promotion and health education interventions related to environmental health issues.

Appropriateness of ecological theory in answering the objective

The core focus of the ecological theory is on the interaction between and interdependence of factors throughout and across all stages of a health issue. For instance, there are five levels of influence of health-related behaviors and circumstances for explaining the fundamental concept of the ecological perspective. These levels include individual or intrapersonal factors, interpersonal factors, community factors, public policy factors, and institutional or organizational factors [13]. Ecological theory allows a study to address the community levels problems by taking into consideration the public and institutional policy factors, social networks and social norms.

With respect to the first principle of ecological theory, it should be observed that ecological psychology rapidly reflects for stressing the objective elements of the environment than those that are subjective. On the contrary, this dichotomy distorts the pragmatist and empiricist roots of these theories, which highlight the existence of specific realities using transactions among the people and the environment over a specific period. The objective or subjective differentiation may be better comprehended as a continuum varying from observable to any trained observer using perceptible only to the individual being studied throughout this perspective [14]. For instance, some phenomena of interest to environmental psychological researchers might be examined regardless of recourse to the experience of individuals as when public records are associated with mortality and morbidity.

Other phenomena may depend majorly in the subject of personal experience; for instance, the association of perceived social support to subjective well-being over time. Each example has strengths and limitations that affect conclusions regarding person and environment relationships. The relationship between person and environment can be advanced if hypotheses are stated by evidence; multiple methodologies are used onto the problem of interest; variables are measured at different scales; quantitative techniques are used for evaluating the relative significance of different complicated interdependencies; and examining the temporal dynamics of these relationships.

Appropriateness of ecological theory in supporting the methods

One of the advantages of using ecological theory is that sampling considerations are usually focused on the representativeness of the individuals as compared to the representativeness of the setting. Indeed, both these concerns are essential for the external validity of studies [15]. However, the range or variance of the environmental variable of interest is the more serious issue that has not received enough attention throughout ecological theory.

A case study is one of the appropriate methods that allow for the apprehension of the contextual aspects that affect the understanding of the phenomenon in question. Similarly, a multiple case study focuses both on the way in which it shows itself in each of the case units that make up the study, and on the phenomenon under analysis. The similarities and differences between the cases become essential elements to be considered at the time of analysis. The field diary is an important instrument, specifically in a case study methodology, for recording participant observations ethically and technically essential [16]. Ethically, the description of the field research team encouraged reflection on the developed procedures with the children, which made the bias of their presence in the field more transparent. Technically, it generated data that offer greater understanding of situational and daily aspects, which help interpretation of the findings derived from formal data collection processes.

Exposure estimation is another under-appreciated environmental sampling issue in ecological studies. There is very poor exposure estimation for many of the physical environmental psychologists’ study. People are rarely exposed to the same level of an environmental factor. Individuals move in and out of buildings and neighborhoods usually use variable means of transport such as public transport, walking, car, and elevator. People usually occupy multiple spaces even within the built environment. However, estimation of the role of environmental factors does not take appropriate account of such spatial and temporal variation in human-environmental exposures in human behavior.

Appropriateness of ecological theory in supporting the findings

There is a little confusion in applying quantitative methods in ecological papers such as univariate and multivariate analysis of variance, and ordinary least squares regression. This is due to the fact that all these techniques are assumptions of the General Linear Model (GLM). The GLM is based on a set of principles that may not always be satisfied when modeling associations between the human behavior and physical environment. Furthermore, the GLM can only address a restricted range of conceptual issues of interest in ecological perspective research [17].

The core aspect of an ecological approach, analytically, the physical and social contexts play an essential role in understanding the person, while individual characteristics affect individual experiences and behaviors in the environment. In particular, the study of individuals in context is involved in many of the research questions in ecological studies. The use of GLM is both inadequate and limited when a research question reflects on the individual and contextual determinants of individual consequences [18]. In these conditions, GLM is inadequate as these models believe that the observations are statistically independent of each other. In a cross-sectional study, the statistical tests of the significance of different explanatory variables are falsified when the observations are not independent [19]. In a longitudinal research, the observations are not apparently independent from one another and GLM is limited in its selections of modeling a longitudinal data. Furthermore, GLM processes are limited as they distort individual differences with respect to the environmental context and focus on an average response.

Appropriateness of ecological theory in supporting the recommendations

From an ecological perspective, a core aspect of research in ecological theory must take into account the hierarchical nature of the contexts and settings throughout the individual behavior and experience happens both longitudinally and cross-sectionally. The hierarchical nature of ecological psychology requires appropriate attention to the theoretical and measurement specification of the context, and consequences at the individual level. Therefore, following recommendations are proposed:

· The focus of ecological studies should be on both the space and time. It is important to consider temporal factors that interact predictability and duration of environmental exposure, and the timing of environmental influences over the life course.

· Well-developed theoretical models are important to recognize fundamental aspects along with an appropriate specification of salient procedures in preliminary qualitative description.

· Environments must be sampled with adequate variability for testing theory sufficiently. This suggestion is specifically salient if examiners are interested in mediators or moderators.

· Interest in moderators of environmental influences necessitates appropriate sampling of settings for assuring that the hypothesized moderator is not strongly associated with the setting itself.

· Significant attention has to be paid to the concern of self-selection within and across the environments both as a potential issue and theoretical issue for the explanation of research outcomes.

· New measurement approaches are required that enable the modeling of these multiple aspects, since environmental contexts comprise of several aspects having potentially different behavioral implications.

· Longitudinal research designs must be provided serious consideration for offering tests of critical theoretical perspectives both analytically and substantively wherever possible.

Ecological perspective researchers must protect against over-reliance on self-reports of the physical environment of human environment relations in empirical investigations. The dynamic and complex nature of human environment relationship needs multi-methodological evaluations over time.

## Conclusions

Health promotion strategies can benefit a large number of people, not just individual. Furthermore, policy level interventions include passive interventions that do not need maintained effort on the part of an individual. On the contrary, behavioral change models focus on individual change and reflect active interventions that need sustained and voluntary effort for achieving behavioral outcomes. Therefore, the integration of ecological models becomes essential in examining limitations such as income, geographic mobility, and education when planning health promotion programs. This theory is also important in including social, cultural, and physical factors that affect health consequences, which include factors such as social cohesion, behavioral patterns, emotional wellbeing, genetic heritage, and developmental maturation.
